# Hexaacetyl-chitohexaose, a chitin-derived oligosaccharide, transiently activates citrus defenses and alters the feeding behavior of Asian citrus psyllid

**DOI:** 10.1038/s41438-019-0158-y

**Published:** 2019-06-08

**Authors:** Qingchun Shi, Justin George, Joseph Krystel, Shujian Zhang, Stephen L. Lapointe, Lukasz L. Stelinski, Ed Stover

**Affiliations:** 1 US Horticultural Research Laboratory, USDA/ARS, Fort Pierce, FL 34945 USA; 2 Citrus Research and Education Center, University of Florida, Lake Alfred, FL 33850 USA

**Keywords:** Pattern recognition receptors in plants, Plant signalling

## Abstract

Plants have a perception system triggered by pathogen and pest signals to initiate defense. These signals include evolutionarily conserved molecules from microbes and insects termed pathogen/herbivore-associated molecular patterns (PAMPs/HAMPs). Here we showed that hexaacetyl-chitohexaose (HC), an oligosaccharide from chitin, a structural component in insect exoskeletons and fungi cell walls, upregulated defense-associated genes *WRKY22*, *GST1*, *RAR1*, *EDS1*, *PAL1* and *NPR2*, and downregulated *ICS1* at 1 h after HC treatment in Sun Chu Sha mandarin leaves. The effect was transient as defense gene transcriptional changes were not observed at 18 h after the treatment. Electrical penetration graph (EPG) recordings were used to study the feeding behavior of Asian citrus psyllid (ACP) following the HC treatment. ACP is the hemipteran vector of *Candidatus* Liberibacter asiaticus (CLas), the pathogen associated with huanglongbing (HLB). Adult ACP displayed reduced intercellular probing, reduced xylem feeding count and duration, and increased non-probing activity on HC-treated citrus compared to controls. During an 18-h recording, percentage for total duration of xylem ingestion, phloem ingestion, intercellular probing were lower, and the percentage of non-probing behavior was higher in HC-treated leaves than in controls. In host-selection behavior studies, HC treatment did not alter the attractiveness of citrus leaves under light or dark conditions. In addition, ACP feeding on HC-treated leaves did not show differences in mortality for up to 10 day of exposure. In summary, we report that HC induced a transient defense in citrus and an inhibitory effect on ACP feeding but did not affect host selection or the insect fitness under the tested conditions.

## Introduction

The bacterial disease Huanglongbing (HLB) or citrus greening is endemic and significantly impacting the citrus industries in the United States and worldwide^1–3^. The associated pathogen, *Candidatus* Liberibacter asiaticus (CLas), is a Gram-negative, intracellular bacterium residing in the phloem cells of the plant host. CLas is transmitted by the Asian citrus psyllid (ACP, *Diaphorina citri*), an insect capable of acquiring the bacterium from infected trees and transmitting to healthy ones through phloem feeding. Citrus infected by CLas display typical HLB symptoms, including leaf blotchy mottle and yellow shoots. A severe infection can result in stunted and declining trees along with fruit drop and/or small misshaped fruits that significantly reduce citrus yield and commercial value. Understanding the HLB pathosystem has been hindered by the fastidious nature of the bacterium, which makes studies related to bacterial pathogenicity and development of control strategies challenging. Genetic resistance to the bacterium is lacking in cultivated *Citrus*; however, HLB tolerance has been identified in some citrus species and relatives, evidenced by reduced developmental impairment by CLas infection^4–9^. Therefore, it is important to monitor and control the insect vector as major part of disease management.

Insecticidal suppression of ACP has been the major tactic for insect control. In Florida, the application of broad-spectrum pesticides during the winter has been recommended as it can suppress ACP populations and reduce need for insecticide during citrus flushing when beneficial insects are present^10^. During leaf flushing, additional sprays of selective insecticides further reduce ACP adults though nymphs are affected to a lesser degree^11^. Broader efficacy testing of insecticides with different modes of action may enhance insecticide rotation to avoid resistance development^12^. Biological control may serve as a sustainable ACP management strategy. Naturally occurring predators, including ladybeetles (Coleoptera: Coccinellidae), lacewings (Neuroptera: Chrysopidae), predatory mites (Acari: Phytoseiidae), and syrphid flies (Diptera: Syrphidae), have been shown to contribute to ACP mortality^13–16^. The exotic parasitoid *Tamarixia radiata* Waterston (Eulophidae), has been imported into several countries, including the United States, and it has been widely established in Florida and California citrus growing areas^16–18^. Utilization of host resistance is another long-term strategy for insect pest management. Studies have shown that the citrus relative *Poncirus trifoliata* displayed relatively low ACP colonization, and possesses both antixenosis and antibiosis resistance characteristics^19–22^, which may contribute to breeding for ACP resistance.

Plants exploit an arsenal of structural, chemical and biochemical defenses against herbivore attacks. Morphological features, such as trichomes, spines, cuticles, thorns, and lignified cell walls, can directly deter the feeding of herbivores^23–25^. Plant secondary metabolites that either function as phytoanticipins or phytoalexins render plant tissue toxic or impart an antifeedant effect^26^. Among the plant defensive chemicals, phenols^27–29^, flavonoids^30^, and tannins^31^ are well documented secondary metabolites with roles in insect defense. Ingestion of various defensive proteins disrupts insect digestion and contributes to plant protection. Examples include lectins which are carbohydrate-binding proteins that survive insect digestive systems and are insecticidal^32^; proteinase inhibitors (PIs) that bind insect digestive enzymes and impair protein digestion^33^, disrupting insect growth, development, reproduction, and even survival^34–37^; and anti-oxidative enzymes such as peroxidases (PODs), polyphenol oxidases (PPOs), and lipoxygenases (LOXs) which have roles in insect deterrence via either direct toxicity or host defense activation^38^.

Plants can perceive microbial and insect molecules as danger signals and mount effective defense against invasions. Studies on interactions with phytopathogens have established that plants have a layered innate immune system which responds to different microbial elicitors^39^, and these early signaling events are similar to those induced by insects^40–43^. Well-studied pathogen-associated molecular patterns (PAMPs) include chitin from fungal cell wall, epitopes of bacterial flagellin (flg22) and elongation factor Tu (efl18), which can elicit plant defenses that protect from subsequent pathogen infections^44–46^. Although the identities of many insect-derived elicitors (herbivore-associated molecular patterns/HAMPs) remains unclear, the host defenses can be triggered by oral secretions, saliva, and fluid from oviposition^47^. The recognition of such insect elicitors affects the outcome of plant-insect interactions. For example, leaf infiltration with crude extracts from green peach aphids (GPA) triggered innate immune responses resembling those against phytopathogens and resulted in increased insect mortality^48^. Treatment of plants with the protein extracted from GPA saliva-induced expression of defense genes and local resistance that reduced insect productivity^49^.

A previous investigation of citrus defense against bacterial pathogens indicated that flg22-associated PAMP-triggered immunity (PTI) played an important role in resistance to citrus canker^50^. In this study, we showed that hexaacetyl-chitohexaose (HC), an oligosaccharide derived from chitin that has been established as the elicitor in other plants^51,52^, induced the expression of defense-associated genes in Sun Chu Sha mandarin as an herbivore-associated molecular pattern, a response similar to the one triggered by flg22. Using the electrical penetration graph (EPG) method, the feeding behavior of ACP was monitored and HC treatment displayed an antifeedant effect against ACP. Further studies indicated that HC treatment did not appear to modify host attractiveness, nor the survival of psyllids as observed by reduced feeding activities in the EPG analysis. The impact of HC-induced transient defense on the citrus–ACP interactions is discussed.

## Results

### HC-induced transient activation of defense genes in Sun Chu Sha mandarin

**Fig. 1:**
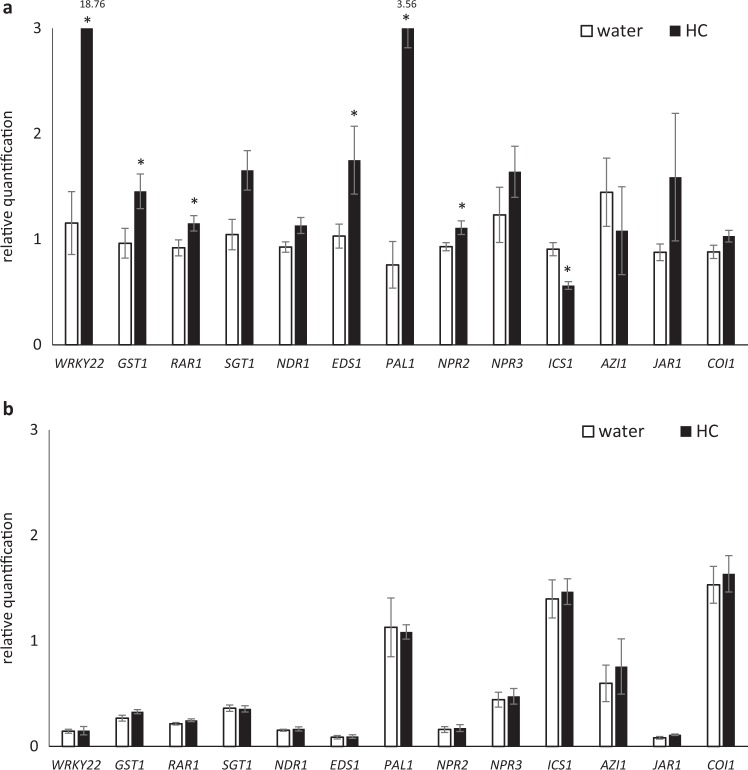
The effect of HC on the expression of citrus defense-associated genes at 1h (a) and 18h (b) after the treatment. (**a**) and 18 h (**b**) after the treatment. HC solution at 100 µg/mL was infiltrated into young fully expanded Sun Chu Sha mandarin leaves and infiltration of water was used as control. Gene relative quantification values were normalized with the endogenous gene *GAPDH* and calculated in reference to a randomly selected control sample. The genes analyzed are indicated on the *x* axis. An asterisk stands for significant difference between HC treatment and control for the gene studied (*p* < 0.05). Bars are means ± standard error (*n* = 3)

A previous investigation indicated that citrus varieties, including Sun Chu Sha mandarin, were sensitive to bacterial flg22 and can activate the expression of a suite of defense-associated genes representing key functional nodes of defense pathways^50^. These genes were PTI markers (*WRKY22* and *GST1*)^53^, PTI and effector-triggered immunity perception and signaling genes (*RAR1*, *SGT1*, *NDR1*, and *EDS1*)^54–57^, genes encoding salicylic acid (SA) signaling (*NPR2*, *NPR3*, and *AZI1*)^58–60^ and biosynthesis (*PAL1* and *ICS1*)^61,62^, and jasmonic acid (JA) perception and metabolism genes (*COI1* and *JAR1*)^63,64^. Here, the effect of HC on the expression of citrus defense-associated genes was studied in Sun Chu Sha mandarin at 1 h and 18 h after treatment. Results indicated significantly increased expression of *WRKY22*, *GST1*, *RAR1*, *EDS1*, *PAL1*, and *NPR2*, and reduced expression of *ICS1* at 1 h in the plants treated with HC (Fig. 1a). This effect was not observed at 18 h after the treatment and none of the genes studied showed expression level changes compared with water controls at this time point (Fig. 1b).

### HC modified ACP feeding behavior in Sun Chu Sha mandarin

**Fig. 2:**
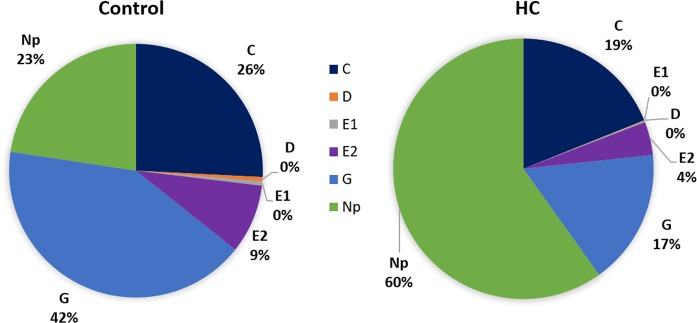
The effect of hexaacetyl-chitohexaose (HC) treatment on the total duration of *D. citri* feeding activities during interactions with Sun Chu Sha mandarin leaves. Young fully-expanded leaves were infiltrated with 100 µg/mL of HC solution or water as the Control. Psyllids were placed on citrus leaves 1 h after infiltration and feeding behavior was recorded by EPG for an 18 h duration. The total duration of intercellular probing (**C**), phloem penetration (**D**), phloem salivation (**E1**), phloem ingestion (**E2**), xylem ingestion (**G**), and non-probing (**Np**) are indicated by percentage in the pie charts. The data represent the average of 10 biological replicates for each treatment

ACP behavior was studied on HC-treated vs. control citrus leaves to determine if HC-induced defense gene activation corresponded to plant resistance to insects. Insect feeding behavior was monitored using EPG recordings, which generated waveforms representing specific feeding activities, including intercellular probing (C), phloem penetration (D), phloem salivation (E1), phloem ingestion (E2), xylem ingestion (G), and non-probing (Np). EPG recording was conducted for 18 h starting at 1 h after HC or control treatment, which were the same time points studied for defense gene induction. For the 18 h recording period, the mean number of waveform bouts (count), mean duration, and total duration for each type of feeding activity are shown in Table 1. Results indicated that there was significantly less intercellular probing (C) for psyllids feeding on HC-treated leaves than the controls (Table 1). Significant reductions in the count, mean, and total duration of xylem feeding activities were observed for HC-treated leaves compared with controls (Table 1). Psyllids feeding on HC-treated leaves spent significantly longer durations of time on non-probing (Np) activities than on the controls (Table 1). Over the total duration of feeding activity studied (18 h), HC treatment resulted in 60% of total time in non-probing (Np) compared with 23% in controls (Fig. 2). The percentages of xylem ingestion (G) (17% vs. 42%), phloem ingestion (E2) (4% vs. 9%), and intercellular probing (C) (19% vs. 26%) were lower in the HC treatment than in controls.

**Table 1:** The number of waveform bouts (count), mean duration and total duration (count × mean duration) of feeding activities of *Diaphorina citri* adults on young Sun Chu Sha mandarin leaves, over 18 h following hexaacetyl-chitohexaose (HC) treatment

Treatment					
Waveform	Variable	HC	Control	Chi square-value	*P*-value
C	Count	24.2 ± 6.1	45 ± 7	5.12	0.02*
	Mean duration (min)	9.5 ± 1.1	6.7 ± 1.2	2.93	0.09
	Total duration (min)	203.8 ± 49.6	279.2 ± 37.1	2.71	0.10
D	Count	2.0 ± 0.6	6.1 ± 2.6	0.34	0.56
	Mean duration (min)	0.8 ± 0.3	0.6 ± 0.2	0.24	0.62
	Total duration (min)	1.7 ± 0.4	6.2 ± 2.8	0.24	0.62
E1	Count	2.0 ± 0.6	6.1 ± 2.6	2.40	0.13
	Mean duration (min)	0.7 ± 0.2	0.8 ± 0.3	0.33	0.56
	Total duration (min)	2.0 ± 1.0	5.7 ± 1.9	1.05	0.33
E2	Count	1.0 ± 0.3	2.7 ± 1.2	0.02	0.89
	Mean duration (min)	24.3 ± 15.3	41.9 ± 32.3	0.05	0.82
	Total duration (min)	44.8 ± 31.0	94.6 ± 44.1	0.05	0.86
G	Count	5.3 ± 1.2	20.8 ± 2.0	12.5	0.0004*
	Mean duration (min)	35.5 ± 4.6	23.7 ± 4.7	3.95	0.04*
	Total duration (min)	182.2 ± 47.1	451.7 ± 64.6	7.71	0.005*
Np	Count	17.2 ± 4.2	18.8 ± 4.1	0.95	0.33
	Mean duration (min)	75.7 ± 41.2	20.2 ± 7.1	6.32	0.01*
	Total duration (min)	646.2 ± 49.1	244.1 ± 51.3	11.6	0.0007*

The feeding activities studied were intercellular probing (C), phloem penetration (D), phloem salivation (E1), phloem ingestion (E2), xylem ingestion (G), and non-probing (Np). Data were analyzed by Kruskal–Wallis test (*n* = 10, *α* = 0.05) using JMP without differentiating insect sex (v. 10, SAS Inc, Cary, NC, USA)

### The effect of HC on ACP host-selection behavior in Sun Chu Sha mandarin

**Fig. 3:**
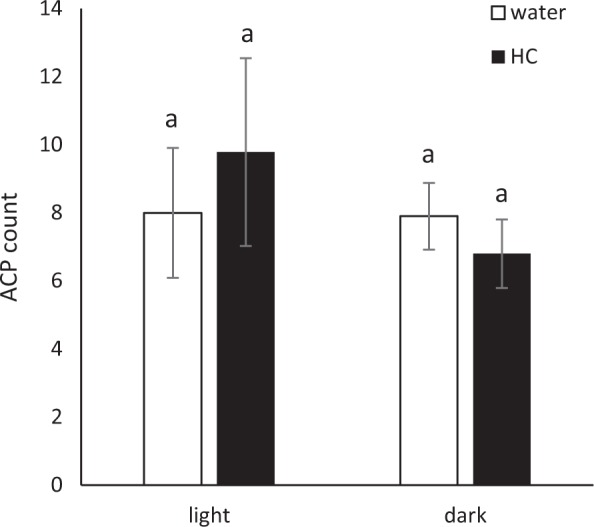
The effect of hexaacetyl-chitohexaose (HC) on host selection in Sun Chu Sha mandarin. Young fully expanded leaves were infiltrated with 100 µg/mL of HC or water as controls. The injected leaves were detached from plants and placed in vials with an opening on top. Each setup is a cage containing 50 adult *D. citri* with two vials inside for 24 h. The number of psyllids that entered each vial was recorded. The experiments were carried out 14 times in a chamber with light (*n* = 14) and 20 times without light (*n* = 20). Statistical significance was determined by Student’s *t* using JMP Genomics. Bars are means ± standard error

The choice assay comparing HC- and control-treated leaves was conducted to determine if HC-induced defense affected host selection, perhaps through modification of volatile releases. The experiments were compared in both light and dark conditions. There was no significant difference between the numbers of ACP entering vials containing HC-treated leaves or control leaves under either light or dark conditions, indicating no effect of HC treatment on host preference (Fig. 3).

### The effect of HC on ACP mortality in Sun Chu Sha mandarin

**Fig. 4:**
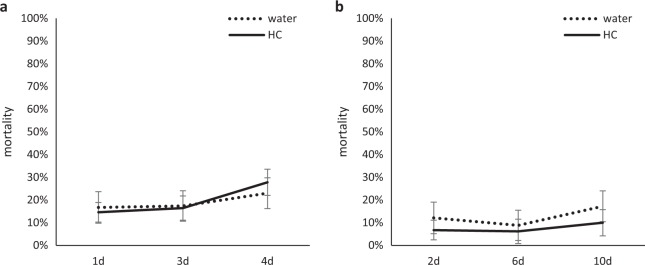
The effect of HC treatment on the mortality of D. citri on the detached (a) and attached leaves (b) of Sun Chu Sha mandarin. (**a**) and attached leaves (**a**) of Sun Chu Sha mandarin. Young fully expanded leaves were infiltrated with 100 µg/mL of HC or water as controls. In the detached leaf assay, injected leaves were removed from the plants and each exposed to 10 adult psyllids for 4 days (*n* = 10 per time point and treatment). For a longer period of observation, attached leaves on greenhouse plants received HC or water treatment and were enclosed in a Petri dish with 10 psyllids/leaf, and the mortality was monitored for 10 days (*n* = 8 per time point and treatment). Statistical significance was determined by Student’s *t* using JMP Genomics. Bars are means ± standard error

Leaf infiltration of HC resulted in transient induction of defense-associated genes and reduced xylem and phloem feeding by ACP. To determine if this effect had any long-term influence on ACP fitness, the mortality of psyllids feeding on HC-treated citrus was compared with those feeding on the control plants. In detached leaf assays, the ACP mortality was counted at 1, 3, and 4 days after the treatment, and there was no difference between HC and control treatments (Fig. 4a). Longer period of observation of ACP mortality was performed using intact plants. Results showed no difference in mortality between HC and control treatments at 2, 6, or 10 days after leaf infiltrations (Fig. 4b).

## Discussion

Chitin is comprised of N-acetylchitooligosaccharides and is the major constituent of fungal cell walls, insect exoskeletons and egg shells of nematodes. Earlier studies demonstrated that chitin or fungal cell wall extracts, applied to plants, induced plant cell physiological changes, including lignification, phytoalexin production, membrane depolarization, and extracellular alkalization. These observations indicate there is a chitin perception system in plants which activates defenses against fungal pathogens^65–68^. Further research revealed that both monocot and dicot plants can perceive chitin through a membrane-located receptor and kinase protein complex^69^, leading to signal transduction and activation of defense-related genes^70,71^. In this study, chitin treatment of Sun Chu Sha mandarin induced differential expression of citrus defense-related genes, including *WRKY22*, *GST1*, *RAR1*, *EDS1*, *PAL1*, *NPR2*, and *ICS1*, at 1 h after the treatment (Fig. 1a), and this effect was not observed at 18 h after treatment (Fig. 1b). The quick activation of defense genes suggests citrus has a similar chitin perception system that can rapidly respond to this elicitor. In the responses of Arabidopsis to PAMPs, chitin-triggered transcriptional reprogramming was the strongest between 0.5 and 3 h after exposure^70^, and flg22-induced defense gene activation from 0.5 to 5 h after exposure^53^. Citrus gene induction at 1 h but not at 18 h after treatment indicates HC induced a transient defense in Sun Chu Sha mandarin and the timing appears to be consistent with those in model plants.

In a previous study, flg22 from the citrus canker causing bacterium *Xanthomonas citri* ssp. *citri* upregulated the expression of *WRKY22*, *GST1*, *EDS1*, *NDR1*, *RAR1*, *SGT1*, *PAL1*, and *NPR3* in Sun Chu Sha mandarin^50^, a pattern highly similar to that induced by HC (Fig. 1a), indicating shared defense pathways between flg22- and chitin-induced defenses in citrus. Among the HC-induced genes, *WRKY22* and *GST1* are considered early markers for flg22-initiated PTI^53^ and *WRKY22* was also shown to be induced by chitin^46^; *RAR1* and *EDS1* are important genes for R gene-mediated pathogen resistance (ETI)^56,72^, basal and nonhost resistance related to PTI^73–75^; *PAL1* and *ICS1* encode key enzymes for two different pathways for biosynthesis of the defense hormone SA^61,62^, and plants respond to chitin in a SA-dependent manner^70^. In addition, *trans*‐cinnamic acid, the production of which is catalyzed by *PAL1*, is a precursor for biosynthesis of lignin and antimicrobial secondary metabolites that contribute to pathogen resistance^61,76,77^. Application of chitin to leaves increases activity of the enzyme PAL and enhances total phenolic content^78^. Taken together, these results suggest that chitin activates defense pathways in citrus that is similar to other plant species, and may have a role in citrus resistance to pathogens and possibly to insect pests.

Studies on chitin-triggered defenses have focused on the plant-pathogen interactions^79^, where evidence has shown that perception of chitin can confer resistance to biotrophic and necrotrophic fungal pathogens^46^. Only a few investigations have addressed chitin-induced insect resistance as an insect-derived elicitor, although crude insect extracts that may contain chitin were shown to induce PTI-like responses in Arabidopsis and leaf infiltration resulted in higher insect mortality^48^. In this study, EPG was employed to investigate ACP feeding activities as influenced by chitin treatment. Results from 18 h recordings indicated ACP had reduced feeding activity on HC-treated leaves, including lower counts of intercellular probing (C), and count and duration of xylem feeding (G) (Table 1). HC treatment led to reduced duration of xylem ingestion (G), phloem ingestion (E2), and intercellular probing (C) and greatly increased non-probing (Np) time (Fig. 2). This suggests HC-induced defense in Sun Chu Sha mandarin has an inhibiting effect on ACP feeding. This effect may result from rapid defense initiation upon chitin perception, SA-mediated defense and/or production of inhibitory secondary metabolites^80^, which were reflected as transcriptional activation of genes in the related pathways in this study (Fig. 1a). It is likely that additional plant defense mechanisms, that are dependent or independent to what were investigated in this study, may have contributed to the alteration of ACP feeding as a result of HC treatment, which is worthy of analysis through systematic approaches such as transcriptomics and proteomics. However, in a previous study it was discovered that formic acid, a carboxylic acid produced as a breakdown product of citrus volatiles^81^, induced the same set of defense-associated genes in citrus within several hours of gaseous exposure, and EPG showed reduced ACP feeding on the treated plants^82^. These results suggest a good correlation between expression activation of these genes and citrus resistance to ACP feeding, and hence supports potential for using transcriptional markers for resistance assessment in citrus.

Additional studies of HC-induced defense on citrus–ACP interactions were conducted using host-selection behavior and mortality assays. Results indicated no significant differences in host selection by ACPs between HC-treated and control leaves, and no host preference was observed in experiments conducted under either light or dark conditions (Fig. 3). Plants activate a wide range of defenses in response to herbivore attack or pathogen infection, resulting in release of volatile organic compounds (VOCs)^83–85^. It was reported that citrus plants infected with HLB were more attractive to *D. citri* than healthy plants, possibly due to higher amounts of methyl salicylate released from infected plants, suggesting an interplay between citrus defense and insect host-selection behavior^86^. In this study, leaves with HC injection did not show change in attractiveness to ACP, suggesting HC-induced local defenses may not be associated with the release of VOCs that modulate host selection. It is possible that the effect from HC treatment of single leaves may not lead to sufficient production of olfactory cues to influence ACP host selection. In addition, the activation of SA-associated defense genes by HC (Fig. 1) implies the initiation of systemic defenses in citrus^87^ which can lead to protection of distant tissues and even neighboring plants from herbivores^85^. Hence it will be interesting to conduct tests at the whole plant scale for effect of chitin on the odorant-mediated ACP host preference and on systemic induction of defense against insects.

To determine if HC-induced feeding inhibition affected ACP fitness, mortality was analyzed on detached leaves within 4 days of treatment, and leaves from intact plants within 10 days of treatment. Results showed no difference in ACP mortality between HC and control treatments at the time points studied (Fig. 4). In the gene expression studies, the effect of the HC treatment occurred at 1 h but not at 18 h after the treatment, suggesting the antifeedant effect is also a short-term effect which may not be strong or long-lasting enough to impact ACP mortality. In the study of flg22-triggered PTI in citrus, leaves infiltrated with flg22 showed reduced pathogenic bacterial growth at 2 and 4 days post inoculation (DPI) but the effect disappeared by 6 DPI^50^, similar to the transient effect HC induced in citrus against ACP and suggesting that ACP may have resumed normal feeding when the effect ceased. In tobacco–green peach aphid interactions, insects fitness was analyzed through nymph feeding of tobacco leaves transiently expressing the effector protein Mp10^88^. Nymphs were fed with Agro-infiltrated leaves for 6 days and moved to newly Agro-infiltrated leaves until the emergence of adults and next generation of nymphs, where reduced fecundity (number of nymphs produced per adult) was observed due to plant defense induced by Mp10^88^. Hence, a similar assay may be useful to investigate if HC-induced defense in citrus may lead to a meaningful insect resistance phenotype.

In conclusion, this study showed that HC is an herbivore-associated molecular pattern in citrus that triggered transient induction of defense-associated genes, including *WRKY22*, *GST1*, *RAR1*, *EDS1*, *PAL1*, *NPR2*, and *ICS1*. The HC treatment resulted in an inhibitory effect on ACP feeding behavior, including reduced intercellular probing and xylem feeding and increased duration of non-probing activity, in an 18-h EPG recording. Moreover, HC-treated citrus leaves did not have altered host attractiveness regardless of presence or absence of light. Finally we showed that a HC-induced, transient antifeedant effect did not lead to increased ACP mortality.

## Materials and methods

### Plant and insect materials

Sun Chu Sha mandarin (*Citrus reticulata* Blanco) plants were grown from seeds in cone containers under greenhouse conditions. Seedling plants <1 year old with multiple young fully expanded leaves were selected for experiments. The plants were watered and acclimated in a walk-in chamber (26 °C, 65% relative humidity, 14-h light and 10-h dark cycle) 1 day prior to experiments. The colony of Asian citrus psyllids (*Diaphorina citri*) were maintained at the insectary of USDA-ARS US Horticultural Research Laboratory using *Citrus macrophylla* as host plants. The sex ratio of the colony was about 1:1. Adult psyllids at 8–10 days old were collected from the cage via vacuum and used for experiments.

### HC treatment

Chitin-derived HC (Megazyme Inc., Chicago, IL, USA) was used as an elicitor. HC was dissolved at 10 mg per 1 mL of deionized water, autoclaved for 10 min and centrifuged for 5 min at maximum speed with a benchtop centrifuge. The supernatant was collected and lyophilized into powder. A 50 µg/mL water-based solution was prepared for experiments. Leaf infiltration of the HC solution was used and water infiltration was used as the controls.

### EPG recordings

EPG was performed using a DC-monitor, GIGA-8 system (EPG-Systems, Wageningen, the Netherlands) to record the feeding activities of adult ACP on HC or water injected citrus leaves. For each setup, a single psyllid was tethered to recording equipment using a gold wire at 1.5 cm long and 25 µm diameter (Sigmund Cohn Corp., Mt. Vernon, NY) attached with silver conducting glue (Ladd Research Industries, Burlington, VT), and then settled on a leaf at the area of treatment (at 1 h after treatment). To complete the circuit, a second electrode (ground electrode) was inserted into soil at the base of the plant. EPG recordings were conducted in a climate-controlled chamber (26 °C and 60–65% humidity) for 18 h under lighted conditions. For each treatment, a total of 10 setups were subject to EPG (*n* = 10). Waveforms were classified by visual inspection according to previous reports into six feeding states^89^: intercellular probing (C), phloem penetration (D), salivation at phloem (E1), phloem ingestion (E2), xylem ingestion (G), and non-probing (NP). The waveforms were annotated in the Dataq Waveform Browser (Dataq Instruments Inc., Akron, OH). The number of waveform bouts (count), mean duration and total duration (count × mean duration) for individual waveforms were analyzed by Kruskal–Wallis test (*α* = 0.05) using JMP without differentiating insect sex (v. 10, SAS Inc, Cary, NC, USA).

### Expression analysis of citrus defense-associated genes

Multiple young fully expanded leaves from each Sun Chu Sha mandarin plant were infiltrated with HC solution or water. The treatment used a 1-mL insulin syringe with a needle to inject solution into the abaxial surface of leaves until the leaf was saturated. Three plants per treatment were used as the biological replicates. Leaf samples were collected at 1 h and 18 h after infiltration for RNA extraction. Samples were put in liquid nitrogen and stored in a −80 °C freezer. Total RNA was isolated by TriZol reagent (Invitrogen, Carlsbad, CA, USA) combined with on-column DNase treatment and purification with an RNeasy Plant Mini Kit (Qiagen, Gaithersburg, MD, USA). The cDNA was synthesized using a QuantiTect Reverse Transcription kit (Qiagen) and diluted to 5 ng/µL concentration. Quantitative real time PCR using SYBR green reagent (Thermo Fisher Scientific, Waltham, MA) was performed amplifying from 10 ng of cDNA template. Primers of citrus defense-associated genes were referenced from a previous study^50^. The comparative Ct (ΔΔCt) method was used to calculate the relative quantification of each gene and the *citrus glyceraldehyde-3-phosphate dehydrogenase C2* (*GAPC2*) was used as the endogenous gene for normalization^90^. Statistical significance was determined by nonparametric method (*α* = 0.05) using R (The R Foundation for Statistical Computing, Vienna, Austria).

### Insect mortality assays

For the detached leaf assay, HC or water treated leaves were removed from plants and petioles were placed in 0.5-mL centrifuge tubes filled with water and sealed by Parafilm. Each leaf was put in a 50-mL plastic centrifuge tube with a mesh top, and 10 psyllids were released to feed on the leaf under constant light. The number of dead psyllids was recorded at 1, 3, and 4 days after the setup. For the intact plant assay, HC or water treated leaves were enclosed by a transparent Petri Dish with the edge sealed by Parafilm. Each leaf was fed on by 10 psyllids under constant light. The mortality was recorded at 2, 6, and 10 days after the setup.

### Insect host-selection assay

For each setup, one HC infiltrated and one water infiltrated citrus leaf were removed from the plant immediately after treatment. The petioles were placed in water in 0.5-mL centrifuge tubes sealed with Parafilm. Each leaf was enclosed in a non-transparent vial with a hole on the cap (diameter of 3.8 mm). The two vials were placed in a small cage where 50 adult psyllids were released subsequently. The number of psyllids entering each vial was recorded after 24 h. The assay was repeated 14 times under constant light and 20 times in dark in a controlled environment chamber (26 °C, 65% relative humidity).
